# Impact of Viral Diseases on the Livestock Sector in Bangladesh

**DOI:** 10.1155/vmi/5492206

**Published:** 2025-11-06

**Authors:** Md. Salauddin, Md. Ahanaf Ajmaeen Khan, Azri Rahmati, Md. Golzar Hossain, Masaru Shimada, Sukumar Saha

**Affiliations:** ^1^Department of Microbiology and Public Health, Khulna Agricultural University, Khulna 9202, Bangladesh; ^2^Department of Microbiology and Hygiene, Bangladesh Agricultural University, Mymensingh 2202, Bangladesh; ^3^Department of Molecular Biodefense Research, Graduate School of Medicine, Yokohama City University, Yokohama 236-0004, Japan

**Keywords:** Bangladesh, disease control, economic burden, livestock sector, viral diseases, zoonosis

## Abstract

Viral diseases pose a significant threat to Bangladesh's livestock sector, resulting in substantial economic losses and impeding overall growth. These infections disrupt animal productivity, undermine food security, and place financial strain on farmers. This review provides a comprehensive analysis of the major viral diseases impacting livestock in Bangladesh. The cumulative burden of viral diseases jeopardizes a sector that contributes 1.54% to the national GDP. Factors such as uncontrolled animal movement and trade, along with climate change, exacerbate disease transmission and increase the risk of outbreaks. The economic repercussions extend beyond production losses to include rising food prices and serious public health concerns stemming from zoonotic transmission and antimicrobial resistance. Effective mitigation requires integrated control strategies, including widespread vaccination, strengthened biosecurity, and robust disease surveillance. Enhancing veterinary infrastructure and aligning with international disease control standards will improve market access and ensure sector sustainability. Long-term resilience will depend on coordinated efforts among government agencies, private stakeholders, and international partners to safeguard food security and rural livelihoods.

## 1. Introduction

Viral diseases pose a significant threat to Bangladesh's livestock industry, resulting in severe economic losses and long-term consequences for trade, public health, and food security. Livestock contributes approximately 1.54% to the national GDP and 13.62% to total agricultural output; however, frequent disease outbreaks continue to hinder the sector's development and resilience [1]. These infections not only reduce the production of meat, milk, and eggs but also escalate treatment costs, elevate mortality rates, and trigger trade restrictions. Given Bangladesh's dependence on livestock and poultry farming for rural employment and nutrition, addressing the impact of viral diseases is a national priority.

Among the most economically damaging diseases, foot-and-mouth disease (FMD) remains a persistent challenge, affecting cattle, sheep, and goats. It causes substantial productivity losses, with an estimated annual economic burden of US$60 million [2]. The impact is particularly pronounced in dairy production, where crossbred and indigenous cows incur average losses of Tk. 4,454,924.68 and Tk. 418,454.36, respectively [2]. Peste des petits ruminants (PPR) significantly affects small ruminants, accounting for approximately US$25 million in annual losses [3]. Since its emergence in 2019, lumpy skin disease (LSD) has further intensified the economic burden, contributing to estimated losses of US$91.33 million across affected districts, especially in Mymensingh and Gaibandha [4]. This review hypothesizes that viral diseases in Bangladesh's livestock sector create cascading impacts that extend beyond direct economic losses to affect public health, food security, trade competitiveness, and long-term sectoral sustainability, requiring integrated management approaches.

The poultry sector has also faced significant setbacks due to recurrent viral outbreaks. Avian influenza (AI) (H5N1) prompted mass culling during the 2007-2008 outbreak, resulting in economic losses of US$388.58 million [5]. Newcastle disease (ND), an endemic viral pathogen, causes an average annual loss of US$288.49 million [6]. Other viral infections—such as infectious bursal disease (IBD), Marek's disease, and fowl pox—collectively result in multimillion-dollar losses annually [7–9]. Infectious bronchitis virus (IBV) alone is responsible for US$34–47 million in losses each year, with a prevalence of up to 41.67% in Tangail and an associated 30%–50% reduction in egg production [1, 3]. Waterfowl diseases, including duck viral hepatitis and duck plague, also contribute significantly, with mortality rates ranging from 46.7% to 95% and financial losses estimated at US$11–17 million annually [10, 11] (Department of Livestock Services [DLS], 2022).

Beyond direct economic impacts, viral diseases pose broader threats to international trade and public health. Frequent outbreaks limit Bangladesh's ability to comply with international sanitary standards, constraining exports of livestock and poultry products [12]. Zoonotic viruses, particularly avian influenza, present serious health risks to humans, while the overuse of antibiotics to manage secondary infections accelerates the development of antimicrobial resistance (AMR) [3, 13]. Climate change further exacerbates these risks by altering viral survival patterns and extending the transmission seasons of pathogens such as FMD, ND, and AI [14–16].

Addressing these multifaceted challenges requires a comprehensive disease control strategy. Strengthening vaccination programs, enhancing farm-level biosecurity, and expanding disease surveillance systems are essential steps toward reducing outbreak frequency and severity. In parallel, investments in veterinary education, infrastructure, and scientific farming practices will bolster national preparedness [17, 18]. Collaborative efforts among government agencies, private stakeholders, and international partners are vital to developing sustainable livestock policies, improving trade readiness, and modernizing veterinary systems [19]. Such efforts are crucial not only for stabilizing the livestock sector but also for securing long-term economic resilience and food security in Bangladesh. The primary objectives of this comprehensive review are to (1) systematically analyze the economic burden of major viral diseases affecting Bangladesh's livestock and poultry sectors; (2) examine the interconnected impacts on public health, food security, and international trade; (3) assess the influence of climate change on viral disease patterns and emergence; (4) evaluate current disease control and prevention strategies; and (5) identify knowledge gaps and provide recommendations for sustainable livestock disease management in Bangladesh.

## 2. Methodology

This review adopted a comprehensive narrative approach to evaluate the impact of viral diseases on livestock in Bangladesh, with a focus on associated economic losses and mitigation strategies. A systematic search was conducted using electronic databases, including PubMed, PubMed Central, and Google Scholar. Additional sources included reports from international organizations such as the World Health Organization (WHO), the World Organisation for Animal Health (WOAH), and the Food and Agriculture Organization (FAO).

The search strategy combined relevant keywords—such as “viral diseases,” “livestock industry,” “economic losses,” “Bangladesh,” “disease impact,” and “mitigation strategies”—along with specific disease names to capture a broad and targeted scope.

The literature search was limited to publications from 2008 to 2024 to ensure the inclusion of up-to-date and relevant findings. More than 300 publications were initially identified. After a rigorous screening process, 102 documents were selected for full review, including 90 peer-reviewed articles, 5 international agency reports, and 7 agricultural economic reports. Ultimately, 71 references were cited in this review.

Inclusion criteria focused on studies directly addressing viral diseases in Bangladeshi livestock, with data on economic losses, disease prevalence, and mitigation efforts. Studies not related to Bangladesh or not addressing viral diseases were excluded. Data extraction emphasized disease-specific prevalence, associated economic impacts, and effects on various livestock species.

This structured approach enabled a thorough synthesis of current knowledge on the economic and epidemiological burden of viral diseases in Bangladesh's livestock sector. The findings provide an evidence-based foundation for analysis, discussion, and policy recommendations.

## 3. Livestock Distribution in Bangladesh

The livestock sector plays a critical role in Bangladesh's economy, contributing to food security, rural livelihoods, and national agricultural output. As of 2023, the estimated populations of major livestock and poultry species were cattle (24.86 million), goats (26.95 million), sheep (3.83 million), chickens (319.69 million), and ducks (66.02 million), with the total poultry population reaching approximately 385.70 million (Livestock Economy at a Glance: https://dls.portal.gov.bd/sites/default/files/files/dls.portal.gov.bd/page/ee5f4621_fa3a_40ac_8bd9_898fb8ee4700/2023-07-23-12-04-afbcccb96f8b27d4bab6501aa8c2c2ff.pdf) [20].

### 3.1. Cattle Distribution

Cattle distribution varies significantly across regions, with densities ranging from 19.7 to 361.7 animals per km^2^. The highest concentrations are observed in the Rangpur Division, particularly in districts such as Barguna, Dinajpur, and Thakurgaon, where cattle densities range from 313 to 464 per 1000 people [21, 22]. Approximately 38.6% of Bangladeshi households own cattle, with small-scale farmers accounting for 59.5% of the total cattle population. The Rajshahi Division alone contributes 21.06% to the national cattle stock [23–26].

### 3.2. Goat Distribution

Bangladesh has a goat population exceeding 30.33 million, 90% of which are Black Bengal goats. High goat densities are reported in Bogura, Sirajganj, Gaibandha, and Rangpur. Goat densities range from 12.4 to 359 per km^2^, with an average of 138.6 goats per 1000 people. Small-scale farmers own approximately 75.6% of the national goat population, highlighting the importance of goat farming in supporting rural incomes and nutrition [22–24].

### 3.3. Sheep Distribution

Sheep distribution is more uneven, with a total population estimated at 3.68 million. Regional densities range from 0.3 to 43.0 per km^2^, with the highest concentrations found in seven districts, where densities vary between 21.3 and 46.6 sheep per 1000 people. Only 1.2% of households are engaged in sheep farming. Nevertheless, 75.8% of the sheep population is raised in farm-based production systems, reflecting its growing role in diversified livestock farming [23, 25].

### 3.4. Poultry Distribution

Poultry farming is a dominant component of Bangladesh's livestock sector. Chickens comprise 255.31 million birds—approximately 90% of total poultry—of which 80% are indigenous breeds. Chicken densities range from 105 to 1212 birds per km^2^, with the highest concentrations found in Dinajpur, Bandarban, and Joypurhat districts [27].

The duck population is estimated at 45.12 million, with high densities in areas such as Barguna, Bhola, and Dinajpur. The national average is 306 ducks per 1000 people, and 28.9% of households are engaged in duck farming [26]. Pigeon farming is also widespread in districts including Chuadanga, Jessore, and Kushtia, where densities range from 100.1 to 198.2 pigeons per 1000 people. Although quail farming is less common, it is growing steadily in Dhaka, Gazipur, Mymensingh, and Chittagong, supported by favorable environmental conditions and a well-developed poultry infrastructure [23, 27].

**Figure 1:**
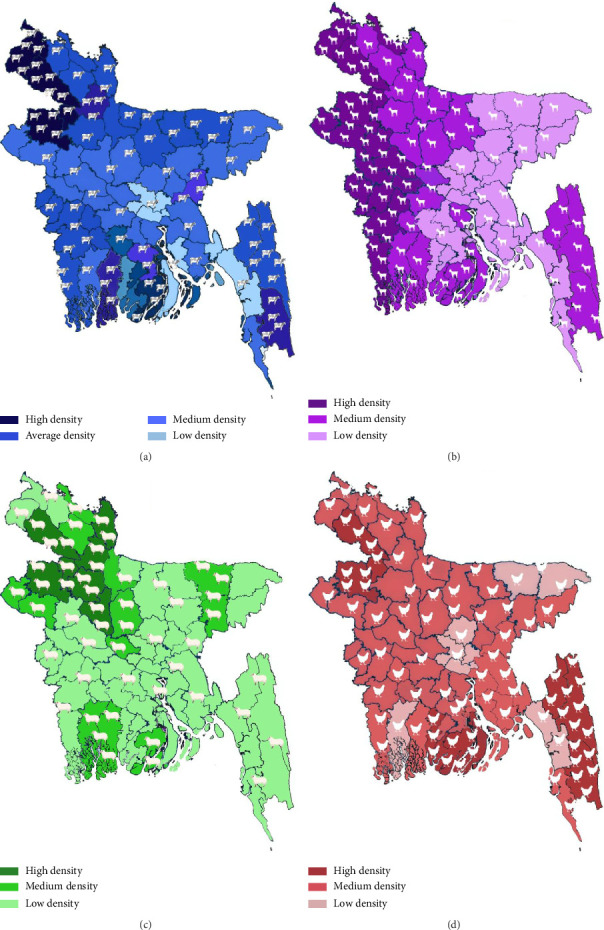
Distribution of livestock and poultry in Bangladesh. (a) Geographical distribution of cattle. (b) Geographical distribution of goats. (c) Geographical distribution of sheep. (d) Geographical distribution of chickens.

Bangladesh's livestock sector holds considerable potential for sustainable development. Strategic investments in infrastructure, modern farming practices, and targeted policy interventions are essential to closing production gaps, improving productivity, and enhancing resilience across species and regions (Figure 1).

## 4. Overview of Viral Diseases in Bangladesh

Bangladesh faces substantial challenges due to the widespread prevalence of viral diseases affecting both the livestock and poultry sectors. Several major viral infections compromise animal health and productivity, including FMD, bovine viral diarrhea (BVD), LSD, PPR, ND, AI, IBD, and rabies. These pathogens are primarily transmitted through direct contact with infected animals, contaminated surfaces, respiratory droplets, or vectors such as mosquitoes and ticks.

The rapid spread and high morbidity associated with these diseases result in considerable economic losses for both farmers and the national livestock industry. Outbreaks diminish animal productivity, increase mortality rates, and incur significant costs related to disease management, vaccination, and veterinary care [28].

## 5. Distribution of Different Viral Diseases of Livestock and Poultry in Bangladesh

Viral diseases remain a persistent challenge to livestock and poultry farming in Bangladesh, posing threats to both animal health and the national economy. The following subsections outline the prevalence, geographic distribution, and epidemiological patterns of key viral diseases, highlighting the urgent need for strengthened biosecurity, disease surveillance, and vaccination coverage.

### 5.1. FMD

**Figure 2:**
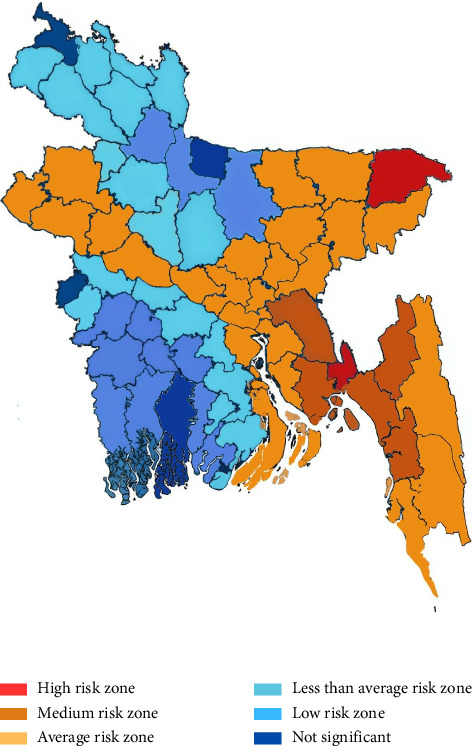
Overall risk zones of foot-and-mouth disease (FMD) in different areas of Bangladesh.

FMD is a highly contagious viral disease primarily affecting cattle, sheep, and goats. It is most prevalent during the winter and early monsoon seasons. Between 2014 and 2017, a total of 548,817 cases were reported, predominantly in cattle. Morbidity was highest among male and older animals (36%). Regionally, Mymensingh reported the lowest number of cases, while Chattogram Division—particularly the districts of Noakhali, Feni, and Lakshmipur—recorded the highest incidence (Figure 2). Unregulated animal movement remains a significant risk factor, underscoring the importance of stringent biosecurity protocols [29].

### 5.2. LSD

LSD emerged as a major threat in 2019, with Bangladesh among the first Asian countries to report cases. The Chattogram region was most affected, exhibiting a morbidity rate of 23%. Other affected districts included Gazipur (1.42%), Narayanganj (0.87%), Dhaka (0.21%), Satkhira (0.06%), and Pabna (0.05%) [30]. The frequent importation of livestock and high cattle density in affected zones contribute to continued disease transmission.

### 5.3. BVD

BVD is prevalent in high-density cattle-farming regions such as Barisal, Kishoreganj, Noakhali, Gopalganj, Bandarban, and Chattogram. The disease leads to reduced productivity, reproductive losses, and increased veterinary expenditures [31].

### 5.4. PPR

**Figure 3:**
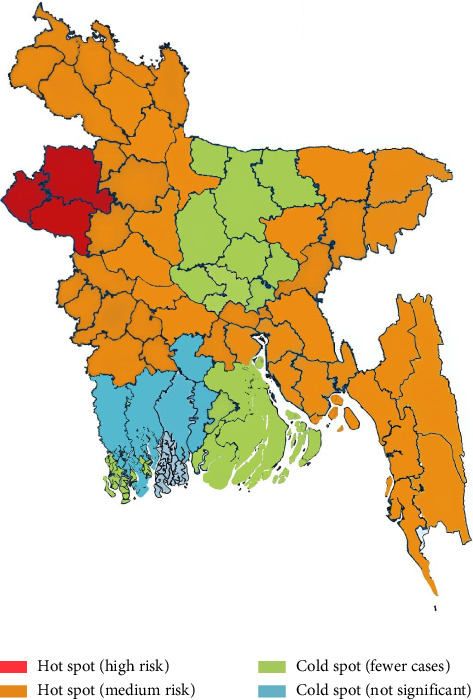
Overall peste des petits ruminants risk zones.

PPR is widespread across Bangladesh. Between 2014 and 2017, 524,805 cases were reported, with incidence peaking during the monsoon season (June). High-risk districts include Rajshahi, Joypurhat, Naogaon, Bogura, Faridpur, Moulvibazar, Sylhet, Noakhali, and Chattogram. In contrast, Barisal, Khulna, Dhaka, and Mymensingh were identified as low-risk areas (Figure 3) [29].

### 5.5. Rabies

Rabies remains a significant zoonotic threat to both humans and livestock. Before 2010, annual rabies-related deaths in humans exceeded 2100. Following the implementation of improved control programs, the number decreased to approximately 200 per year by 2015. Between 2011 and 2022, 724 human rabies deaths were reported. The highest incidence occurred in Dinajpur, Rangpur, and Bogura, where over 13,000 cases were also documented in livestock populations [32].

### 5.6. Cowpox

Although infrequently reported, cowpox remains a potential concern in rural regions of Bangladesh. Outbreaks tend to occur during the winter and rainy seasons and primarily affect dairy cattle, leading to reduced milk production. Zoonotic transmission is possible, with infected individuals experiencing fever, headache, and skin lesions [33].

### 5.7. Sheep and Goat Pox (SGP)

SGP is of considerable economic significance, especially in northern and central Bangladesh. Morbidity rates range between 70% and 90%, with mortality reaching up to 10% in endemic zones and 100% in imported animals. The disease spreads via close contact, aerosols, and contaminated equipment. Seasonal outbreaks are common, and severe infections can result in internal lesions and death. Vaccination remains the most effective control strategy [34].

### 5.8. AI

**Figure 4:**
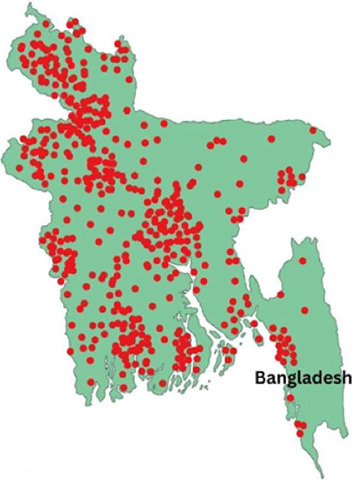
Areas affected by Avian influenza.

Bangladesh has recorded the highest number of Highly Pathogenic Avian Influenza (HPAI) outbreaks in South Asia between 2006 and 2019, with 561 confirmed cases. High-risk zones include Hakaluki Haor, Tanguar Haor, and the Chattogram region, where migratory birds interact with domestic poultry. The H5N1 strain has been detected in 49 out of 64 districts, with live bird markets in Dhaka and Chattogram serving as major hubs of transmission (Figure 4). Carrier species such as pigeons and quail also contribute to viral spread, with prevalence rates of 17.36% and 38.75%, respectively, particularly during the dry season [35].

### 5.9. IBD

IBD is widespread in Bangladesh, particularly in Chattogram and Mymensingh. In Mymensingh, serological surveys revealed that 83.4% of unvaccinated backyard chickens tested positive for IBDV antibodies, indicating high endemicity [36]. The disease causes immunosuppression, leading to increased susceptibility to secondary infections, reduced growth rates, and compromised productivity. Enhanced vaccination strategies are needed to control the spread and limit economic losses.

### 5.10. ND

**Figure 5:**
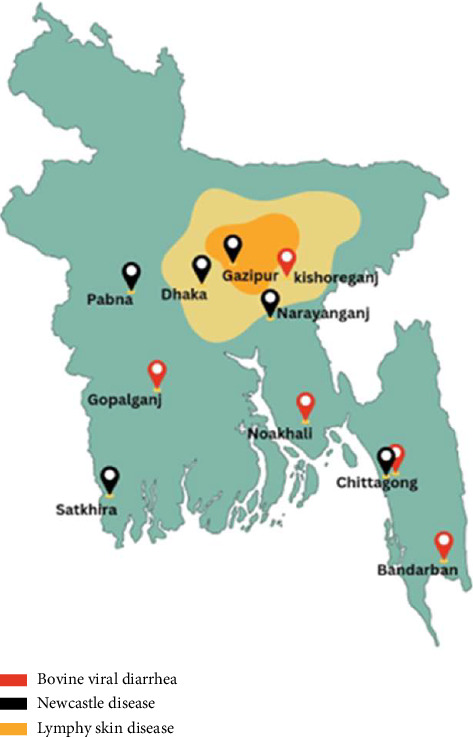
Hot spot of Newcastle disease.

ND is endemic to Bangladesh's poultry sector, with documented outbreaks between 2020 and 2021. Gazipur reported the highest incidence rate (41.67%), while Tangail reported the lowest (20%) (Figure 5) [37]. ND also affects pigeons, with a higher prevalence in the summer (27.24%) compared to winter (21.56%). In quail, the Newcastle Disease Virus (NDV) was responsible for 11.35% of recorded infections, underscoring its cross-species transmission potential and economic impact.

### 5.11. Fowl Pox and Pigeon Pox

Fowl pox is common in poultry-producing districts such as Gazipur, Tangail, Kishoreganj, Narsingdi, and Mymensingh. Similarly, pigeon pox follows a comparable distribution pattern in areas with dense pigeon populations [38]. These diseases, while generally less lethal, contribute to productivity losses through reduced egg production and secondary infections.

### 5.12. Infectious Laryngotracheitis (ILT)

ILT is widespread across Bangladesh, with notable prevalence in regions of intensive poultry farming. Studies report high seropositivity in Gazipur district (81.47% in layer farms), Rajshahi, Rangpur, Chittagong, Khulna, and Barisal divisions (up to 100% in some areas). Lower prevalence was observed in Mymensingh subdistricts (0.4% in Sonali birds) and Chattogram district (17.33% in commercial layers). Seasonal variation is evident, with higher incidence in winter (24%) and among birds aged 10–35 weeks [3, 39].

The continued presence and spread of these viral diseases underscore the urgent need for enhanced disease surveillance, targeted vaccination campaigns, and the implementation of robust biosecurity measures. Strengthening these control mechanisms is essential not only to reduce the economic burden on livestock and poultry producers but also to safeguard public health and food security in Bangladesh.

## 6. Economic Losses due to Viral Diseases in Bangladesh

Viral diseases inflict substantial economic losses on Bangladesh's livestock and poultry sectors, severely hindering productivity and sectoral resilience. Among these, FMD is one of the most economically devastating, with a 2017-2018 study reporting losses of 53.17 million BDT (US$0.63 million) across 850 affected households. Nationally, annual FMD-related losses are estimated at 188.57 billion BDT (US$2.22 billion) [2], reflecting declines in productivity, veterinary expenditures, and labor costs.

LSD, which emerged in 2019, has imposed considerable financial strain. In Mymensingh and Gaibandha districts alone, annual losses were estimated at 7.76 billion BDT (US$91.33 million), primarily due to reduced milk production, increased treatment costs, and cattle mortality [19]. Similarly, BVD contributes approximately 1.28 billion BDT (US$15.09 million) in annual losses, largely resulting from decreased fertility, calf mortality, and milk yield [40].

PPR disproportionately affects small ruminant farmers, leading to an estimated US$25 million in annual losses. Mortality and morbidity rates for goats and sheep reach up to 59% and 75%, respectively [29]. Rabies also imposes a significant economic burden; each case is estimated to cause US$3267.21 in losses due to livestock mortality and lost productivity [41].

The poultry sector faces equally severe challenges. AI, particularly the H5N1 strain, has led to mass culling, supply chain disruptions, and long-term productivity declines. Losses vary across poultry types—broilers, layers, and Sonali chickens—but the impact is widespread [32]. IBD continues to affect poultry health and output, causing losses through increased mortality, reduced egg quality, and elevated veterinary costs, particularly in poorly managed housing systems [36]. ND, endemic in Bangladesh, accounts for annual losses of approximately 24.38 billion BDT (US$288.49 million), with smallholder farmers being most affected [3]. ILT causes severe economic losses through mortality rates up to 50% in adult flocks, reduced egg production, and stunted growth. Infected birds often require culling, while survivors experience prolonged recovery and decreased productivity. Secondary costs include expenses for diagnostics, biosecurity upgrades, and vaccination programs. While exact monetary losses are not quantified in the provided data, the cumulative impact of mortality, reduced output, and management costs significantly strains small-scale and commercial farms alike [3].

Other notable contributors include IBV, which reduces egg production and flock viability, although exact loss estimates remain limited [3, 42]. Chronic viral infections such as Chicken Infectious Anemia Virus (CIAV) and Egg Drop Syndrome-76 (EDS-76) further diminish productivity and increase treatment-related costs [43, 44].

Duck farming—an essential livelihood in Bangladesh's Haor regions—is also highly vulnerable to viral threats. Duck Viral Hepatitis (DVH), caused by Duck Hepatitis A Virus (DHAV), results in high duckling mortality and the collapse of entire farms (BARC Report). Duck plague causes 25%–46.7% mortality, placing millions of smallholder farmers at economic risk [11, 45]. Additionally, duck septicemia affects over two million rural households, with estimated annual losses between 1.2 and 1.8 billion BDT (US$11–17 million) (DLS Report).

Collectively, these viral diseases continue to erode the stability and economic potential of the livestock sector in Bangladesh. The cumulative burden disproportionately impacts smallholder farmers and remains a major obstacle to achieving sustainable agricultural development.

## 7. Impact on the Economy and Livestock due to Viral Diseases

Viral diseases impose a significant economic burden on Bangladesh's livestock sector by reducing productivity, disrupting trade, and undermining rural livelihoods. The financial impact is especially severe for smallholder farmers, who rely heavily on livestock and poultry for income, nutrition, and labor.

FMD substantially decreases milk yield, weight gain, and reproductive efficiency in cattle, sheep, and goats. Mortality rates among cattle range from 5.15% to 12.39%, with calf mortality reaching as high as 21.27% in native breeds. Infected draft animals also impair agricultural productivity, increasing labor costs [2]. The estimated national economic burden of FMD is US$125 million annually, emphasizing the importance of targeted interventions, including vaccination and disease surveillance (World Bank) [46].

LSD has a severe impact on cattle, leading to reduced milk production, weight loss, and decreased marketability. In high-density regions with inadequate biosecurity, morbidity rates can reach 90%, while mortality ranges from 1% to 10% [47]. Khan et al. reported the highest morbidity and case fatality rates for LSDV in the Rajshahi division of Bangladesh, with 71.42% morbidity, 10% case fatality rate, and 7.14% mortality [48]. LSD not only increases veterinary costs and impedes livestock trade but also indirectly affects investment in small ruminant farming, as resources are redirected toward managing LSD outbreaks [49]. The overall economic loss was assessed at BDT 8203.22 crore nationally by Mostari et al. [50].

BVD reduces milk production, fertility, and herd viability. Around 50% of persistently infected calves die within a year, contributing to considerable losses. Congenital abnormalities further lower productivity. Preventive strategies such as diagnostic testing and vaccination entail additional costs, particularly for small-scale producers. Moreover, BVD outbreaks raise the risk of trade restrictions, limiting market access [51].

PPR is a major threat to sheep and goat farming, with morbidity rates ranging from 70% to 90% and mortality rates from 50% to 80% in goats. The disease severely affects rural economies by reducing meat and milk output, raising treatment costs, and creating barriers to both local and cross-border trade [52, 53]. While cattle are less frequently infected, those affected often experience reduced immunity and overall productivity.

Rabies, though less common in livestock, has a high fatality rate and significant economic implications. Each infected animal may result in losses of US$300–700 for cattle and US$100–200 for sheep and goats. Outbreaks lead to 100% livestock mortality, placing heavy financial strain on farmers. Indirect costs arise from prevention measures, market disruption, and decreased consumer confidence in animal products [54–56]. In mixed-farming systems, even poultry farms may face operational challenges during rabies outbreaks.

AI remains one of the most critical threats to Bangladesh's poultry sector. Outbreaks can cause mortality rates exceeding 90%, resulting in estimated annual losses of BDT 4–10 billion [57, 58]. Even surviving birds may exhibit long-term declines in egg and meat production. Government-imposed culling exacerbates supply chain disruptions across hatcheries, feed producers, and distribution networks. Consumer concerns during outbreaks suppress demand and depress market prices, compounding financial losses. AI also indirectly impacts other livestock sectors by raising feed prices, further straining the budgets of cattle, sheep, and goat farmers. Smallholders are particularly vulnerable, facing prolonged recovery periods and increased risk of falling into poverty [57, 59, 60].

ILT poses a critical threat to Bangladesh's poultry sector, which supports livelihoods and food security for millions. The disease disrupts daily production of 120 million eggs and 1363 tons of meat, key contributors to the national economy [3]. Poor biosecurity practices and deep-litter rearing systems exacerbate outbreaks, particularly in densely populated poultry hubs like Gazipur. Vaccination programs using imported live attenuated vaccines are common but may not address local viral strains, leading to recurrent outbreaks. The lack of tailored interventions and reliance on costly imported vaccines further amplify economic burdens, underscoring ILT's role in undermining sectoral resilience and growth [3, 39, 61].

The cumulative economic toll of viral diseases highlights the urgent need for comprehensive disease control frameworks. Key strategies include mass vaccination, biosecurity reinforcement, enhanced disease surveillance, and improved farmer outreach. Without coordinated action, these persistent threats will continue to jeopardize national food security, household incomes, and the sustainable development of Bangladesh's livestock sector.

### 7.1. Duck-Specific Viral Diseases

Ducks represent an essential source of animal protein in Bangladesh, particularly in lowland and Haor regions where aquatic farming systems are prominent. However, the duck farming sector remains highly vulnerable to several persistent viral diseases that threaten both livelihoods and national food security.

### 7.2. Duck Plague (Duck Viral Enteritis)

Duck plague causes mortality rates ranging from 30% to 50%, severely affecting egg and meat production. The cross-border nature of disease transmission complicates the development of effective, regionally appropriate vaccines. Annual economic losses are estimated at US$10–15 million [45, 62].

### 7.3. Duck Septicemia

Duck septicemia is a major concern for over two million rural households engaged in duck farming. The disease raises serious biosecurity and sanitary concerns, restricting poultry and duck exports and limiting Bangladesh's access to regional markets. This directly affects farm profitability and reduces the sector's contribution to agricultural trade (DLS Report).

Recurring outbreaks of duck-related viral diseases continue to undermine food security and economic resilience in vulnerable farming communities. Strengthening veterinary extension services, implementing region-specific vaccination programs, and improving disease surveillance and reporting systems are critical to safeguarding livelihoods and maintaining stable production.

## 8. Contribution of Livestock in National Economy

The livestock and poultry sectors are vital to Bangladesh's economy, serving as key drivers of employment, food production, and rural development. Livestock contributes approximately 1.47%–2% of the national GDP and accounts for over 16% of the country's total agricultural output. The sector has maintained a steady annual growth rate of 3.47% [63]. In 2022-2023, the livestock sector contributed 1.85% to the national GDP at constant prices, with a growth rate of 3.23%, accounting for 16.52% of agricultural GDP, generating a GDP volume of Tk. 73,571 crore at current prices, and providing employment to 20% of the population directly and 50% partly (Source: BBS, 2022-2023; https://dls.portal.gov.bd/sites/default/files/files/dls.portal.gov.bd/page/ee5f4621_fa3a_40ac_8bd9_898fb8ee4700/2023-07-23-12-04-afbcccb96f8b27d4bab6501aa8c2c2ff.pdf).

The poultry industry alone contributes an estimated 1.5%–1.6% of GDP and plays an essential role in both income generation and nutritional security [64, 65]. Together, these sectors provide employment to nearly 20% of the national labor force, with the poultry industry alone supporting approximately six million people through direct and indirect jobs [65].

In addition to their economic importance, livestock and poultry are key sources of protein—including milk, meat, and eggs—that support national food and nutrition objectives. In rural areas, these industries help reduce poverty by diversifying income sources and decreasing reliance on traditional crop farming. They also offer significant opportunities for sustainable development and export growth, particularly in processed poultry products and leather goods.

Given their extensive impact on GDP, employment, and public health, sustained investment in livestock and poultry development is essential for ensuring Bangladesh's long-term economic stability, food security, and resilience in the face of emerging threats [63].

## 9. Impact on Public Health and Food Security

Viral diseases in livestock and poultry, particularly zoonotic infections such as AI, pose serious threats to public health and food security in Bangladesh. These pathogens can be transmitted to humans, especially among individuals in frequent contact with animals, including farmers, butchers, and poultry handlers [66].

In response to recurring outbreaks, the widespread use of antibiotics to prevent secondary bacterial infections has contributed to the rise of AMR. This growing resistance undermines the effectiveness of treatments for both animal and human infections, posing a major public health challenge [9].

Outbreaks of viral diseases disrupt the production and supply chains of essential animal-based foods—meat, milk, and eggs—leading to inflated prices and reduced protein availability. This disproportionately affects low-income populations, who are most vulnerable to food insecurity. Diseases such as FMD, ND, and AI exacerbate these pressures by causing high morbidity and mortality among livestock and poultry [3, 9].

To mitigate these risks, effective disease surveillance, targeted vaccination campaigns, and robust biosecurity protocols are essential. These strategies are not only crucial for containing outbreaks but also for ensuring public health, food availability, and overall economic stability [3, 66].

## 10. Climate Change and Potential Threat of Viral Diseases in Bangladesh

Climate change is increasingly influencing the epidemiology of viral diseases in Bangladesh's livestock and poultry sectors. Rising temperatures, elevated humidity, and altered rainfall patterns have reshaped the ecology of disease transmission, expanding both geographic distribution and outbreak frequency.

Warmer climates and high humidity enhance the survival and transmissibility of pathogens such as foot-and-mouth disease virus (FMDV) and NDV, converting previously seasonal outbreaks into year-round threats [13–15]. Additionally, climate-induced habitat changes have pushed rabies-infected wildlife—such as bats and stray dogs—closer to human settlements, increasing the risk of zoonotic spillover to both livestock and humans. These concerns are especially critical in rural areas with limited access to veterinary and healthcare services [15].

ND outbreaks are increasingly frequent in humid regions, particularly in backyard poultry farms and live bird markets, where environmental control is minimal [41]. AI also remains a persistent public health threat. Highly pathogenic strains such as H5N1 present a pandemic risk, driven by climate-induced viral mutations and the expanding migratory range of wild birds [41, 67]. Altered rainfall patterns have created new wetlands, attracting infected migratory birds and elevating the risk of transmission to domestic flocks [67, 68].

Addressing these emerging challenges requires climate-adaptive disease surveillance systems and proactive control strategies. Integrated One Health approaches that consider environmental, veterinary, and public health dimensions are critical for managing climate-sensitive viral disease risks in Bangladesh.

## 11. Impact of Viral Diseases on Trade and Regulation in Bangladesh

Viral diseases have far-reaching implications for Bangladesh's trade and regulatory environment, particularly within the livestock and poultry sectors. Frequent outbreaks and insufficient disease control measures constrain export opportunities, hinder compliance with international standards, and reduce the country's competitiveness in global markets.

### 11.1. Health and Disease Control Challenges

Bangladesh remains highly vulnerable to transboundary animal diseases such as FMD in cattle and AI in poultry. These diseases pose serious health risks to importing countries, prompting stringent trade restrictions. As a result, recurrent outbreaks have significantly curtailed Bangladesh's access to international markets and diminished its ability to export livestock and poultry products [19].

A critical barrier is the lack of an effective disease surveillance and reporting system. Bangladesh does not yet have sufficient infrastructure for systematic disease monitoring, making it difficult to track outbreaks, control transmission, or satisfy the health certification requirements of importing countries. In the absence of robust tracking mechanisms, Bangladesh struggles to gain international approval for exporting animal-based products [69].

### 11.2. Challenges in Meeting International Trade Standards

Global trade in animal products is governed by strict sanitary and phytosanitary (SPS) standards, which require evidence that products are free of infectious diseases. The high prevalence of viral infections and weak enforcement of disease control programs prevent Bangladesh from meeting these requirements. Consequently, the country is often excluded from high-value export markets for meat, dairy, and poultry [69].

Moreover, the lack of a national traceability system hampers efforts to certify the disease-free status of animal products. Without internationally recognized certification and traceability frameworks, it remains difficult for Bangladesh to demonstrate compliance with global animal health standards, further limiting its market access and credibility [17].

### 11.3. Regulatory and Infrastructure Barriers

Bangladesh also faces substantial regulatory and infrastructural constraints. The country lacks a sufficient number of internationally accredited laboratories for disease testing and certification, resulting in delays and trade rejections. This diagnostic gap undermines efforts to comply with import regulations in destination markets [17].

Additionally, the export of perishable animal products requires reliable cold chain logistics, hygienic processing facilities, and efficient transport systems. However, Bangladesh's underdeveloped infrastructure leads to quality degradation and weakens its position in international trade. Inadequate cold storage and distribution networks further compound the problem [69].

### 11.4. Economic Consequences and Global Trade Relations

Viral disease outbreaks and associated trade restrictions result in considerable economic losses across the livestock and poultry industries. Export revenue declines, investor confidence weakens, and domestic demand drops due to reduced consumer trust in animal products. These cascading effects create market instability and threaten rural livelihoods.

To address these challenges, Bangladesh must enhance its veterinary infrastructure, improve disease surveillance, and align with international trade protocols. Cross-border collaboration with global organizations and trading partners is essential to build capacity, streamline compliance procedures, and expand market access.

Moreover, disease outbreaks frequently trigger heightened regulatory scrutiny. Lengthy bureaucratic processes for export licensing and compliance raise transaction costs and delay shipments, further reducing competitiveness. In contrast to leading livestock-exporting nations, Bangladesh lacks comprehensive trade agreements and simplified export procedures. Without favorable trade policies and structural reforms, the country will continue to face barriers in securing a stable position in global animal product markets [69].

## 12. Viral Disease Control and Prevention Program in Bangladesh

Effective disease control and animal welfare form the cornerstone of Bangladesh's livestock sector. While traditional farming practices remain prevalent—particularly among smallholder farmers raising cattle, poultry, and goats—a gradual shift toward scientifically informed methods is underway. The government, in collaboration with national and international organizations, has implemented several initiatives to enhance disease control and improve animal health standards. Nonetheless, significant challenges persist, especially in rural regions where infrastructure and veterinary services remain limited. To strengthen the national capacity for viral disease control and prevention, the following priority areas should be addressed.

### 12.1. Veterinary Services and Disease Control

DLS plays a central role in the prevention and management of animal diseases. DLS coordinates nationwide vaccination campaigns targeting major zoonotic and economically impactful diseases, including FMD, AI, and rabies. In addition, the department provides training to farmers on disease prevention, vaccination schedules, and improved husbandry practices [18].

### 12.2. Legislation and Policy Development

The Animal Welfare Act of 2019 marked a significant advancement in institutionalizing humane animal treatment. The legislation mandates the provision of adequate nutrition, healthcare, and shelter for all livestock. Complementing this, the National Livestock Development Policy advocates for the adoption of modern farming practices, including enhanced feed quality, improved housing, and the integration of biosecurity protocols to minimize the risk of disease transmission.

### 12.3. Farmer Training and Capacity Building

Training programs, developed in collaboration with NGOs and international partners, aim to educate farmers on animal care, disease prevention, housing management, and hygiene. These programs are particularly valuable in underserved rural areas, where lack of knowledge and access to resources remain major barriers to effective disease control and animal welfare.

### 12.4. Public–Private and International Partnerships

The Government of Bangladesh actively partners with international organizations such as the FAO) and WOAH to develop and implement context-specific disease control standards. These partnerships have supported the dissemination of modern technologies and provided technical training to enhance local veterinary capacity.

### 12.5. Veterinary Education and Workforce Development

To strengthen national disease control capabilities, Bangladesh is expanding veterinary education through specialized universities and institutions offering degrees in veterinary science, animal health, and husbandry. These efforts aim to build a skilled workforce capable of implementing evidence-based practices and responding to emerging health threats within the livestock sector.

While commercial farms have made notable progress in adopting improved disease control measures, rural areas continue to face systemic limitations. Continued investment in veterinary infrastructure, extension services, and farmer training is critical for achieving long-term improvements in livestock productivity, public health protection, and overall animal welfare across the country [70].

## 13. Long-Term Economic Consequences

The economic repercussions of viral diseases in livestock and poultry extend far beyond immediate production losses. Infected animals frequently suffer from reduced productivity, lower milk and egg yields, chronic health conditions, and diminished reproductive efficiency. These persistent effects cumulatively erode the long-term sustainability and profitability of livestock operations.

For example, LSD causes permanent skin lesions and progressive emaciation, resulting in prolonged declines in milk production and reduced market value of affected animals [19]. In the absence of preventive measures, rabies contributes to chronic economic strain through repeated livestock mortality, herd size reduction, and compromised productivity [71]. Similarly, PPR imposes continuous economic burdens by decreasing flock sizes, reducing wool output, and increasing veterinary costs over time [29].

Beyond direct losses, viral disease outbreaks also damage market reputation and erode consumer trust. AI outbreaks can have long-lasting impacts on consumer behavior, reducing demand for poultry products from affected regions [71]. ND leads to persistent egg production declines, which diminish revenue over extended periods [6]. Marek's Disease contributes to chronic flock depletion, compounding mortality-related losses [16], while Fowl Pox reduces both meat and egg yields, leading to gradual reductions in farm profitability without effective management [16].

Addressing these long-term challenges requires sustained investment in disease control infrastructure, including vaccination programs, early diagnostic tools, accessible veterinary care, and farmer education. Strategic and proactive approaches are essential to safeguard the resilience, productivity, and economic viability of Bangladesh's livestock and poultry industries.

## 14. Conclusion

The pervasive impact of viral diseases on Bangladesh's livestock and poultry sectors underscores a critical threat to both economic development and national food security. These pathogens not only cause immediate financial losses but also compromise long-term productivity, disrupt supply chains, and endanger public health, particularly for smallholder farmers who constitute the majority of rural producers.

This review highlights the need for an integrated and multifaceted response to combat viral disease outbreaks. Strengthening biosecurity measures, expanding veterinary infrastructure, and implementing large-scale vaccination programs are vital components of any effective control strategy. Concurrently, investments in research, diagnostic innovation, and farmer education are essential to promote early detection and containment of emerging threats.

Aligning national disease control systems and trade regulations with international standards will further enhance Bangladesh's ability to access global markets and participate in high-value exports. Cross-sectoral collaboration—linking government agencies, private industry, academia, and international organizations—will be critical to achieving these goals.

Ultimately, a proactive and coordinated approach is necessary to build a robust, secure, and resilient livestock sector—capable of sustaining rural livelihoods, improving public health outcomes, and supporting the country's long-term economic growth.

## Data Availability

Data sharing does not apply to this article as no new data were created or analyzed in this study.
